# Supplementary material for: Canine tumor mutational burden is correlated with TP53 mutation across tumor types and breeds
Source: Nat Commun. 2021 Aug 3;12:4670. doi: 10.1038/s41467-021-24836-9 (PMC8333103; doi:10.1038/s41467-021-24836-9)
Supplement: Supplementary file 1 — Supplementary Information [file 41467_2021_24836_MOESM1_ESM.pdf]

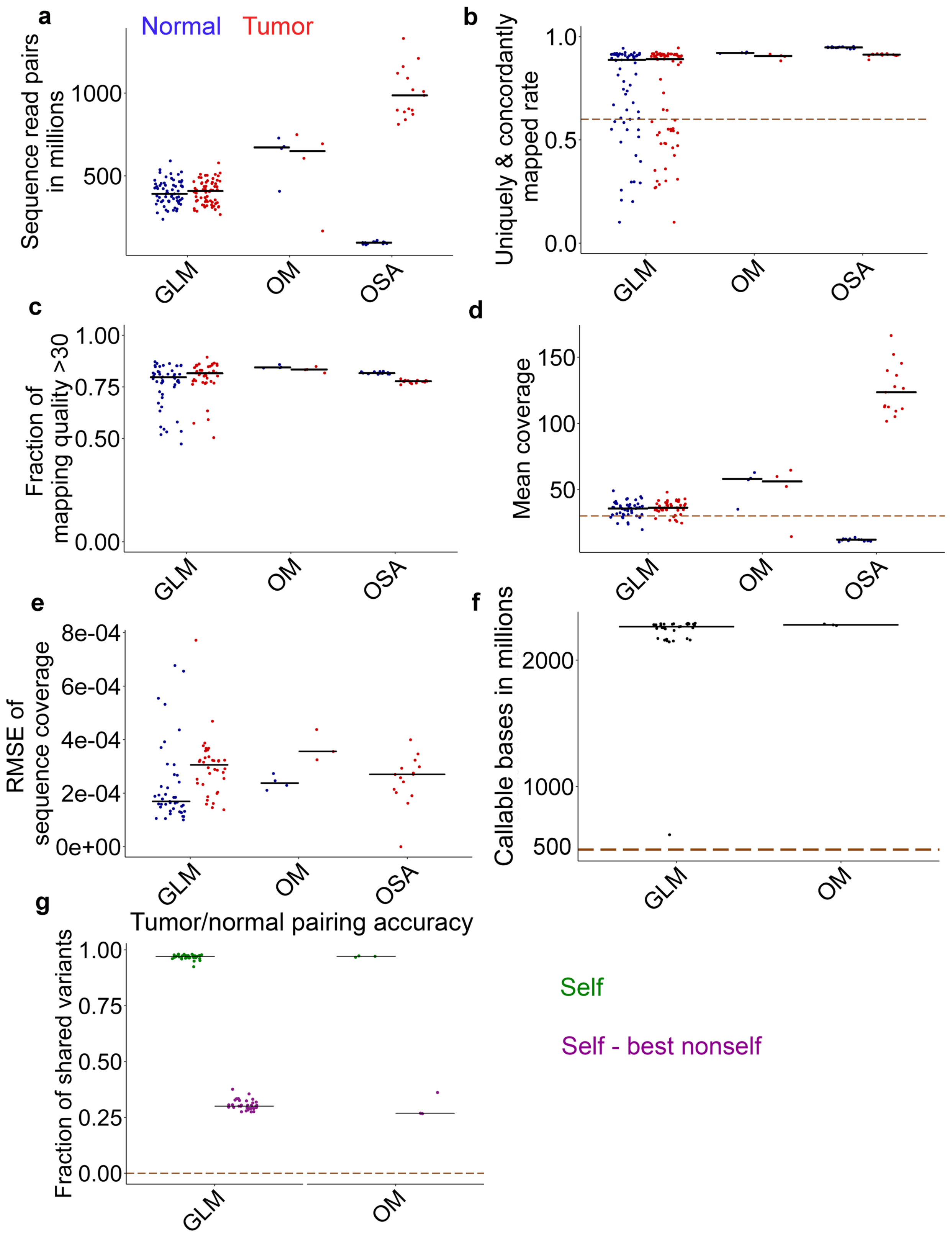

Supplementary Figure 1. Whole-genome sequencing (WGS) data QC. Among 172 paired tumor and normal samples from 86 dogs with published WGS data, a total of 72 samples from 36 dogs passed our QC (Supplementary Data 1). Panels **a-g** are presented as described in panels **a-c**

and **e-h** of Figure 1, respectively. **a-b**) *n* = 67, 4 and 15; **c-d**) *n* = 42, 4 and 15; and **e**) *n* = 36 (33 normal), 4 (3 tumors) and 15 (0 normal samples) independent cases for matched normal and tumor samples of GLM, OM, and OSA respectively. **f-g**) *n* = 33 and 3 independent cases of GLM and OM, respectively. Source data are provided as a Source Data file.

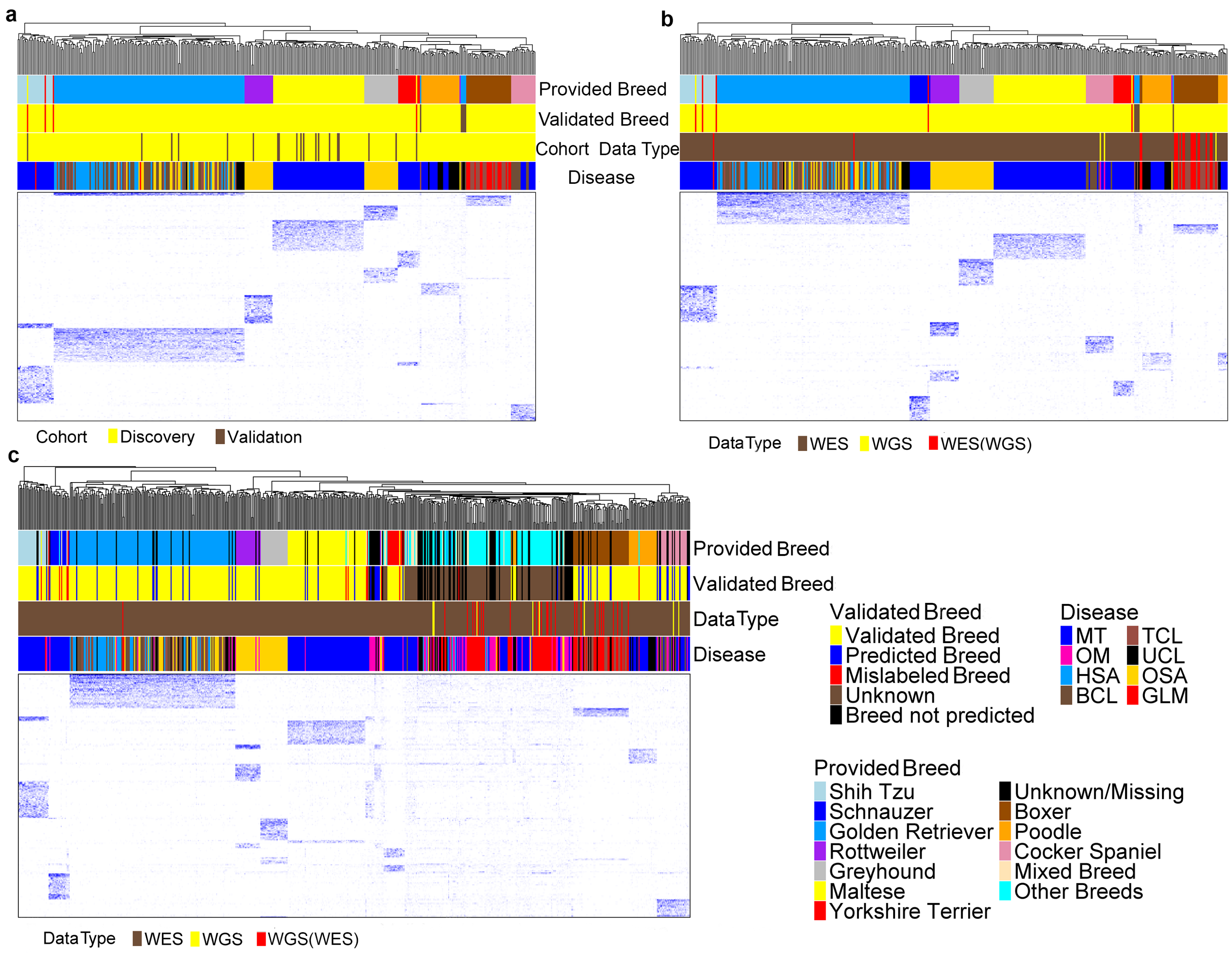
a
Provided Breed
Validated Breed
Cohort
Data Type
Disease
Cohort
Discovery
Validation
b
DataType
WES
WGS
WES(WGS)
c
Provided Breed
Validated Breed
DataType
Disease
DataType
WES
WGS
WGS(WES)
Validated Breed
Validated Breed
Predicted Breed
Mislabeled Breed
Unknown
Breed not predicted
Disease
MT
OM
HSA
BCL
TCL
UCL
OSA
GLM
Provided Breed
Shih Tzu
Schnauzer
Golden Retriever
Rottweiler
Greyhound
Maltese
Yorkshire Terrier
Unknown/Missing
Boxer
Poodle
Cocker Spaniel
Mixed Breed
Other Breeds

Supplementary Figure 2. Corroboration of our breed validation and prediction strategy.

a. The WES dataset is divided into the discovery cohort, which consists of large sample size studies and has 362 dogs, and the validation cohort, which consists of small sample size studies and has 19 dogs (Supplementary Data 2). The discovery cohort has 9 breeds with each having ≥10 dogs, which were used to identify breed-specific germline base substitution and small indel variants (Supplementary Data 2). VAF values of the identified 9 breed-specific variants (5,892 total; see Supplementary Data 2) were then used to cluster the animals from both the discovery and validation cohorts. The image is presented as described in Figure 2.

b. Corroboration with the WGS dataset. Dogs from both WES and WGS datasets were clustered with VAF values of the 10 breed-specific variants (see Figure 2). In the "Data type" bar, the "WES(WGS)" label represents dogs that have both WES and WGS data but were clustered here solely based on their WES data.

c. Corroboration with all 626 dogs from both WES and WGS datasets using the 10 breed-specific variants. All dogs with data that have passed QC shown in Figure 1 and Supplementary Figure 1 were clustered here, including dogs from mixed breeds or breeds with <10 animals. The image is presented as described in Figure 2.

Source data are provided as a Source Data file.

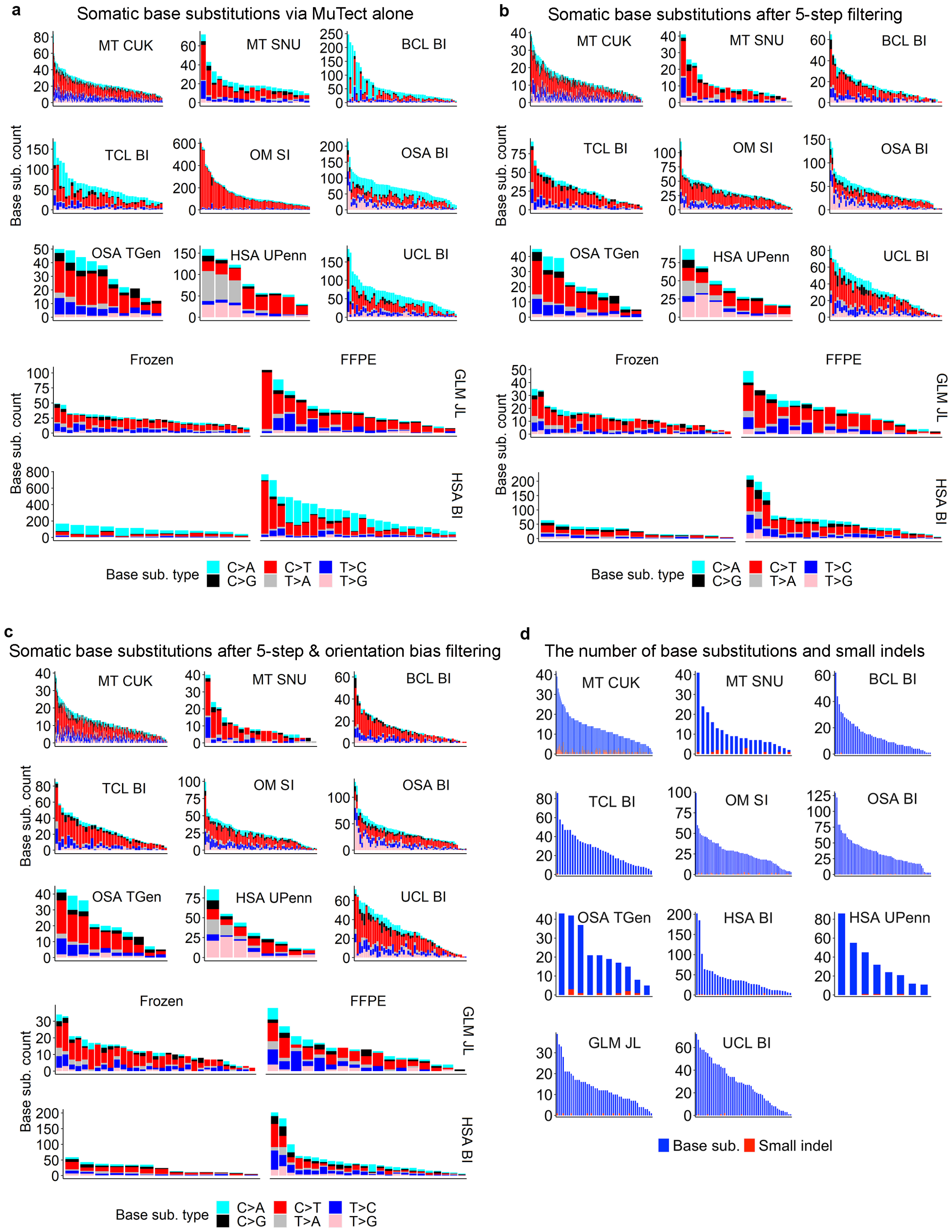

Supplementary Figure 3. Somatic mutation discovery and filtering.

a. Distributions of the six base substitution counts in each tumor of each study called by MuTect. Each study is represented by the tumor type and the institute name. MT: mammary

tumor; GLM: glioma; BCL: B-cell lymphoma; TCL: T-cell lymphoma; OM: oral melanoma; OSA: osteosarcoma; HSA: hemangiosarcoma; UCL: unclassified. CUK: Catholic University of Korea; SNU: Seoul National University; JL: Jackson Laboratory; SI: Sanger Institute; BI: Broad Institute; UPenn: University of Pennsylvania.

b. Distributions of the six base substitution counts in each tumor of each study called by MuTect followed by 5-step filtering[1] (see Methods).

c. Distributions of the six base substitution counts in each tumor of each study called by MuTect, followed by the 5-step filtering[1] and paired-read strand orientation bias filtering (see Methods).

d. Distributions of base substitution and small indel counts in each tumor of each study. Base substitutions were discovered as described in **c**, and small indels were discovered with Strelka (see Methods). The results indicate very few small indels compared to base substitutions.

Source data are provided as a Source Data file.

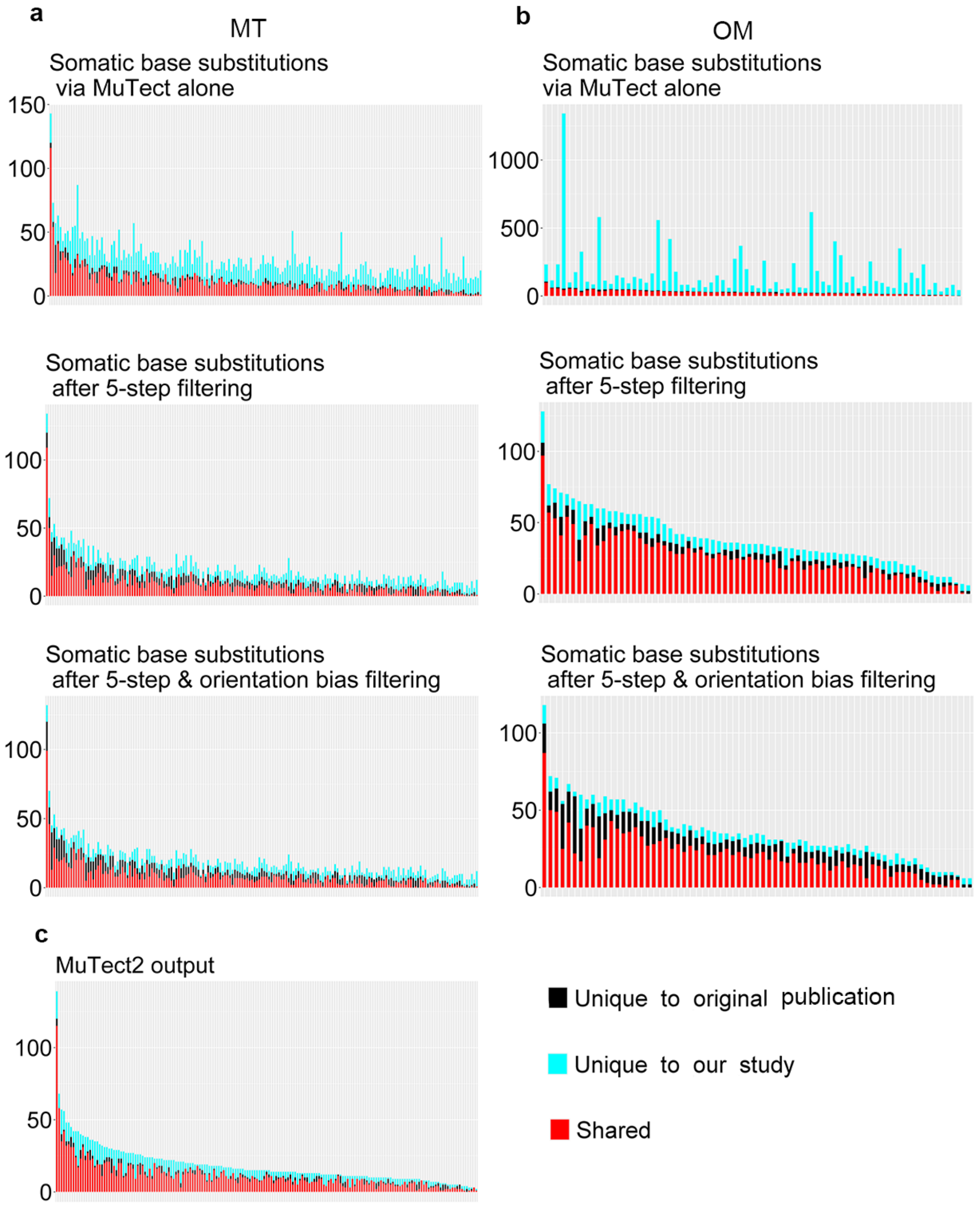

a
MT
Somatic base substitutions via MuTect alone
150
100
50
0
Somatic base substitutions after 5-step filtering
100
50
0
Somatic base substitutions after 5-step & orientation bias filtering
100
50
0
b
OM
Somatic base substitutions via MuTect alone
1000
500
0
Somatic base substitutions after 5-step filtering
100
50
0
Somatic base substitutions after 5-step & orientation bias filtering
100
50
0
c
MuTect2 output
100
50
0
Unique to original publication
Unique to our study
Shared

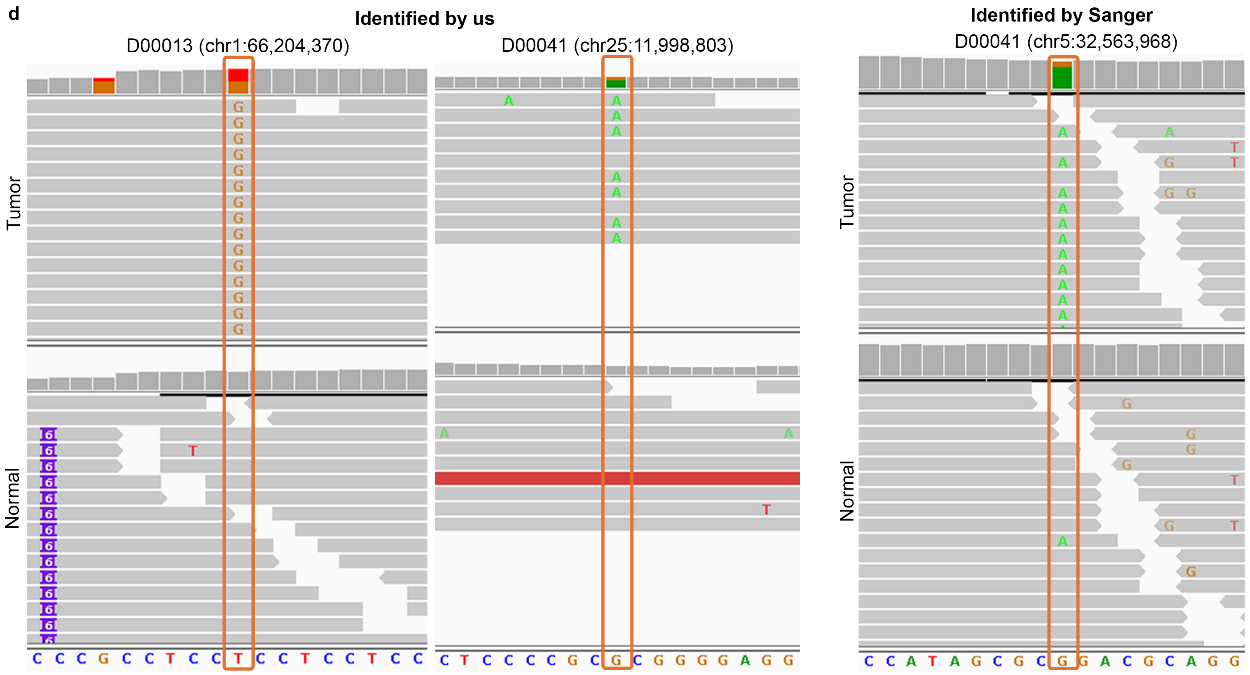

Supplementary Figure 4. Comparison of somatic mutation findings between our study and the original publications.

a-c. Mutations discovered at each of the three steps of our pipeline (see Supplementary Figure 3a-c) were compared to those from the original publications. For each mutation in each sample, the genomic coordinate and the actual mutation, which are published only for mammary tumor (MT)[2] (**a**) and oral melanoma (OM)[1] (**b**), were compared. Distributions of identical and different mutation counts in each sample were plotted. As the original MT publication[2] used MuTect2 instead of MuTect for mutation calling, we also conducted the analyses using MuTect2 and plotted the comparison (**c**).

d. Examples of somatic mutations found only by our study (left two images) or by the original publication (right image). Images are screen shots from the IGV program, with the tumor name provided (e.g., DD00013) and the mutation indicated by an orange rectangle. Source data are provided as a Source Data file.

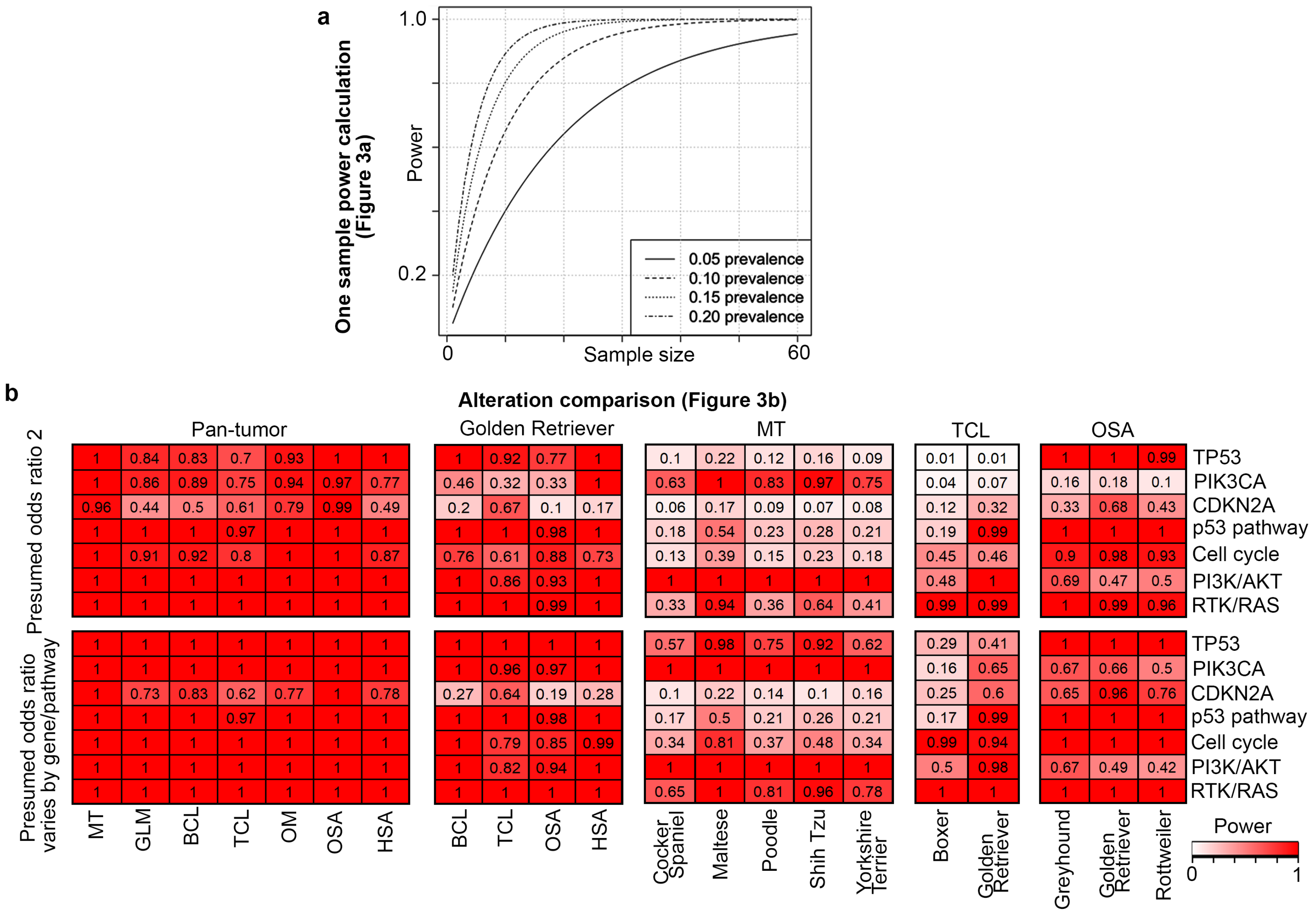
a
One sample power calculation
(Figure 3a)
Power
1.0
0.2
0
Sample size
60
0.05 prevalence
0.10 prevalence
0.15 prevalence
0.20 prevalence
b
Alteration comparison (Figure 3b)
Pan-tumor
Golden Retriever
MT
TCL
OSA
Presumed odds ratio 2
Presumed odds ratio varies by gene/pathway
TP53
PIK3CA
CDKN2A
p53 pathway
Cell cycle
PI3K/AKT
RTK/RAS
1 0.84 0.83 0.7 0.93 1 1 | 1 0.92 0.77 1 | 0.1 0.22 0.12 0.16 0.09 | 0.01 0.01 | 1 1 0.99
1 0.86 0.89 0.75 0.94 0.97 0.77 | 0.46 0.32 0.33 1 | 0.63 1 0.83 0.97 0.75 | 0.04 0.07 | 0.16 0.18 0.1
0.96 0.44 0.5 0.61 0.79 0.99 0.49 | 0.2 0.67 0.1 0.17 | 0.06 0.17 0.09 0.07 0.08 | 0.12 0.32 | 0.33 0.68 0.43
1 1 1 0.97 1 1 1 | 1 1 0.98 1 | 0.18 0.54 0.23 0.28 0.21 | 0.19 0.99 | 1 1 1
1 0.91 0.92 0.8 1 1 0.87 | 0.76 0.61 0.88 0.73 | 0.13 0.39 0.15 0.23 0.18 | 0.45 0.46 | 0.9 0.98 0.93
1 1 1 1 1 1 1 | 1 0.86 0.93 1 | 1 1 1 1 1 | 0.48 1 | 0.69 0.47 0.5
1 1 1 1 1 1 1 | 1 1 0.99 1 | 0.33 0.94 0.36 0.64 0.41 | 0.99 0.99 | 1 0.99 0.96
1 1 1 1 1 1 1 | 1 1 1 1 | 0.57 0.98 0.75 0.92 0.62 | 0.29 0.41 | 1 1 1
1 1 1 1 1 1 1 | 1 0.96 0.97 1 | 1 1 1 1 1 | 0.16 0.65 | 0.67 0.66 0.5
1 0.73 0.83 0.62 0.77 1 0.78 | 0.27 0.64 0.19 0.28 | 0.1 0.22 0.14 0.1 0.16 | 0.25 0.6 | 0.65 0.96 0.76
1 1 1 0.97 1 1 1 | 1 1 0.98 1 | 0.17 0.5 0.21 0.26 0.21 | 0.17 0.99 | 1 1 1
1 1 1 1 1 1 1 | 1 0.79 0.85 0.99 | 0.34 0.81 0.37 0.48 0.34 | 0.99 0.94 | 1 1 1
1 1 1 1 1 1 1 | 1 0.82 0.94 1 | 1 1 1 1 1 | 0.5 0.98 | 0.67 0.49 0.42
1 1 1 1 1 1 1 | 1 1 1 1 | 0.65 1 0.81 0.96 0.78 | 1 1 | 1 1 1
MT
GLM
BCL
TCL
OM
OSA
HSA
BCL
TCL
OSA
HSA
Cocker Spaniel
Maltese
Poodle
Shih Tzu
Yorkshire Terrier
Boxer
Golden Retriever
Greyhound
Golden Retriever
Rottweiler
Power
0
1

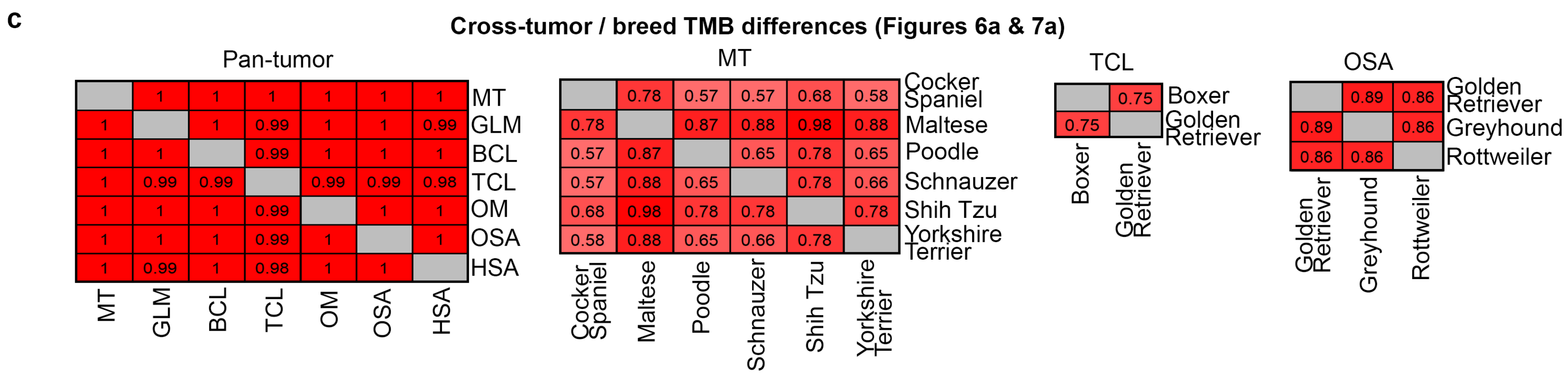
c
Cross-tumor / breed TMB differences (Figures 6a & 7a)
Pan-tumor
MT GLM BCL TCL OM OSA HSA
MT: 1 1 1 1 1 1
GLM: 1 1 0.99 1 1 0.99
BCL: 1 1 0.99 1 1 1
TCL: 1 0.99 0.99 0.99 0.99 0.98
OM: 1 1 1 0.99 1 1
OSA: 1 1 1 0.99 1 1
HSA: 1 0.99 1 0.98 1 1
MT
Cocker Spaniel Maltese Poodle Schnauzer Shih Tzu Yorkshire Terrier
Cocker Spaniel: 0.78 0.57 0.57 0.68 0.58
Maltese: 0.78 0.87 0.88 0.98 0.88
Poodle: 0.57 0.87 0.65 0.78 0.65
Schnauzer: 0.57 0.88 0.65 0.78 0.66
Shih Tzu: 0.68 0.98 0.78 0.78 0.78
Yorkshire Terrier: 0.58 0.88 0.65 0.66 0.78
TCL
Boxer Golden Retriever
Boxer: 0.75
Golden Retriever: 0.75
OSA
Golden Retriever Greyhound Rottweiler
Golden Retriever: 0.89 0.86
Greyhound: 0.89 0.86
Rottweiler: 0.86 0.86

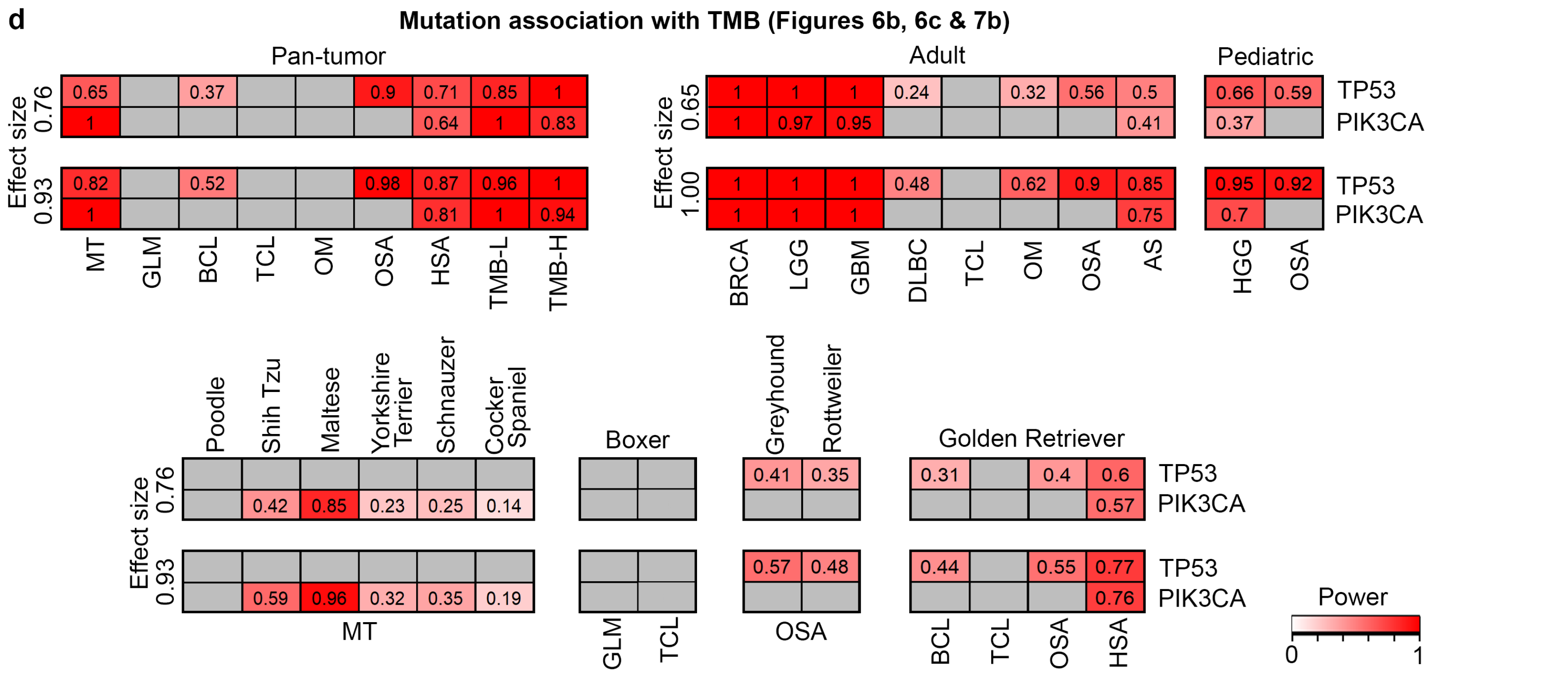
d
Mutation association with TMB (Figures 6b, 6c & 7b)
Pan-tumor
Effect size
0.76
0.93
MT GLM BCL TCL OM OSA HSA TMB-L TMB-H
0.65 0.37 0.9 0.71 0.85 1
1 0.64 1 0.83
0.82 0.52 0.98 0.87 0.96 1
1 0.81 1 0.94
Adult
Effect size
0.65
1.00
BRCA LGG GBM DLBC TCL OM OSA AS
1 1 1 0.24 0.32 0.56 0.5
1 0.97 0.95 0.41
1 1 1 0.48 0.62 0.9 0.85
1 1 1 0.75
Pediatric
HGG OSA
0.66 0.59
0.37
0.95 0.92
0.7
TP53
PIK3CA
TP53
PIK3CA
Poodle Shih Tzu Maltese Yorkshire Terrier Schnauzer Cocker Spaniel
Effect size
0.76
0.93
0.42 0.85 0.23 0.25 0.14
0.59 0.96 0.32 0.35 0.19
MT
Boxer
GLM TCL
Greyhound Rottweiler
0.41 0.35
0.57 0.48
OSA
Golden Retriever
BCL TCL OSA HSA
0.31 0.4 0.6
0.57
0.44 0.55 0.77
0.76
TP53
PIK3CA
TP53
PIK3CA
Power
0
1

Supplementary Figure 5. Power calculation.

a. One-sample power calculation is for the mutation detection power. The power of detecting a mutation within a tumor type or breed is estimated based on the mutation prevalence and the sample size. Simulated curves were generated as described in Methods.

b. Two-sample Fisher exact test power. The power of each Fisher exact test shown in Figure 3b was calculated using: 1) the actual sample sizes of the two groups being compared; and 2) odds ratios as described in Methods.

c. Two-sample Wilcoxon test power for TMB comparison shown in Figures 6a and 7a. Each power was calculated using: 1) the actual sample sizes of the two groups being compared; and 2) an effect size as described in Methods.

d. Two-sample Wilcoxon test power for TMB-gene mutation association analysis shown in Figures 6b, 6c and 7b. The calculation is described in Methods.

Source data are provided as a Source Data file.

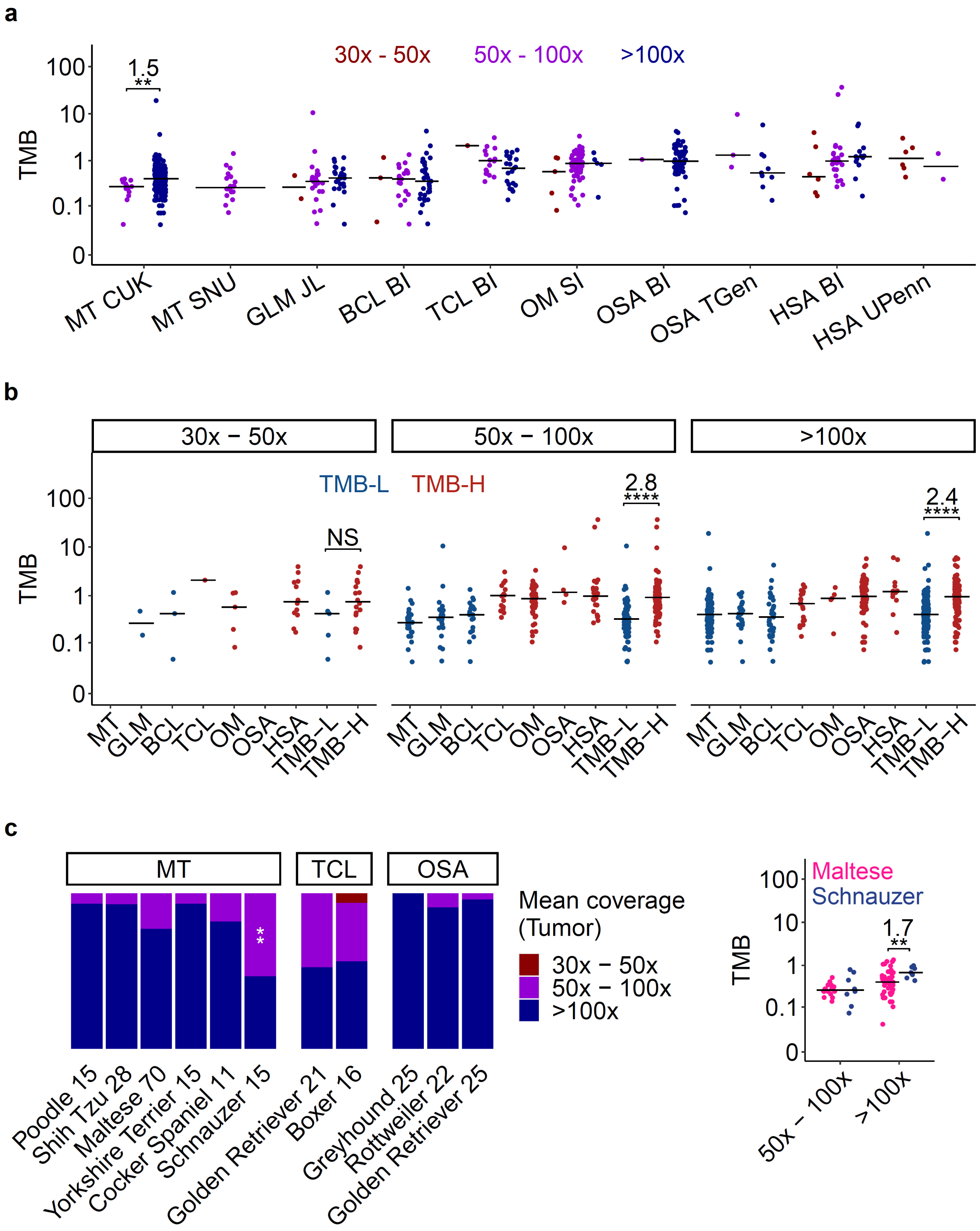

a
30x - 50x
50x - 100x
>100x
TMB
100
10
1
0.1
0
1.5
**
MT CUK
MT SNU
GLM JL
BCL BI
TCL BI
OM SI
OSA BI
OSA TGen
HSA BI
HSA UPenn
b
30x − 50x
50x − 100x
>100x
TMB-L
TMB-H
NS
2.8
****
2.4
****
MT
GLM
BCL
TCL
OM
OSA
HSA
TMB-L
TMB-H
c
MT
TCL
OSA
**
Mean coverage (Tumor)
30x − 50x
50x − 100x
>100x
Poodle 15
Shih Tzu 28
Maltese 70
Yorkshire Terrier 15
Cocker Spaniel 11
Schnauzer 15
Golden Retriever 21
Boxer 16
Greyhound 25
Rottweiler 22
Golden Retriever 25
Maltese
Schnauzer
1.7
**
50x − 100x
>100x

Supplementary Figure 6. TMB at different sequence coverage of 30-50x, 50-100x and >100x.

a. Distribution of tumor mean coverage in each WES study. *n* = 13, 169, 20, 2, 24, 23, 3, 21, 31, 1, 16, 21, 5, 61, 5, 1, 65, 3, 9, 6, 22, 13, 6 and 2 independent tumors from left to right. Two-sided Wilcoxon tests were conducted as indicated; **: $p = 0.007$.

b. TMB comparison at matched sequence coverage among tumor types. *n* = 0, 2, 3, 1, 5, 0, 12, 5, 18, 33, 24, 21, 16, 61, 4, 24, 78, 105, 169, 23, 31, 21, 5, 74, 13, 223 and 113 independent tumors left to right. Two-sided Wilcoxon tests were conducted; NS (not significant): $p = 0.1296$ and ****: $p = 2.3678e-16$ (left) and 7.3985e-18 (right).

c. Distribution of tumor mean coverages among breeds within a study. The left plot indicates that only Schnauzer in MT has a coverage distribution differing from other breeds (**: $p = 0.002$ from two-sided Fisher exact test). The right plot shows that Schnauzer has a higher median TMB than Maltase at 100X coverage; *n* = 16, 8, 54 and 7 independent tumors left to right; **: $p = 0.009$ from two-sided Wilcoxon tests. However, both plots indicate that the sample size of Schnauzer is small.

Source data are provided as a Source Data file.

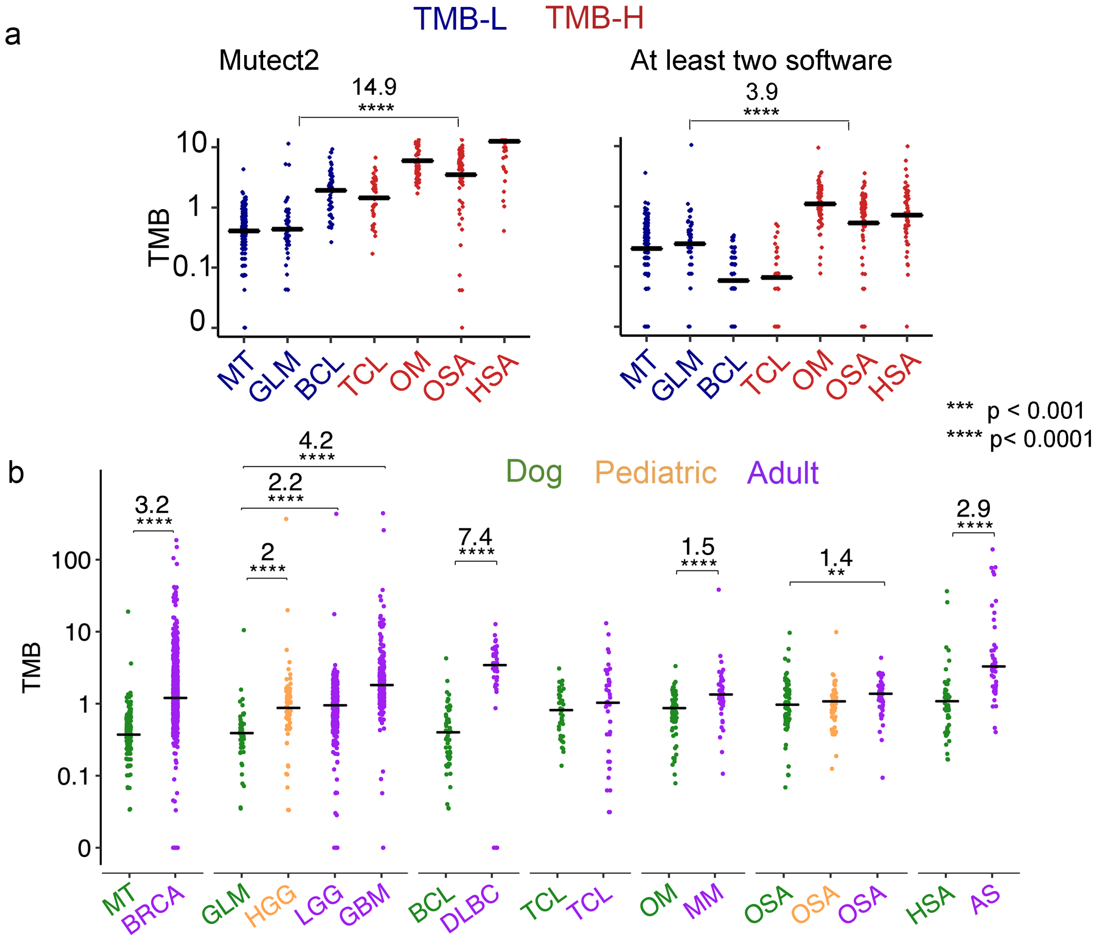

Supplementary Figure 7. Tumor type comparison of TMB obtained by other mutation discovery tools, and dog- human TMB comparison.

a. TMB value of each tumor calculated using somatic mutations called by MuTect2 (left), or at least two of three callers of MuTect2, Varscan2, and LoFreq (right). *n* = 202, 49, 55, 38, 71, 78 and 49 independent tumors from left to right in both plots. Two-sided Wilcoxon tests were conducted, with **** representing *p* = 2.6e-62 and 4.2e-36 from left to right and fold-changes indicated.

b. Dog-human TMB comparison for matched tumor types. Two-sided Wilcoxon test *p* values (**** from left to right representing *p* = 2e-16, 2e-16, 1.6e-15, 2e-7, 1e-10, 2.5e-5 and 6.1e-9 respectively; *** representing *p* = 0.007) and fold changes are indicated for those with significantly different TMB. Abbreviations of tumor types are described in Figures 1,4 and 6. *n* = 202, 1048, 49, 66, 514, 389, 55, 41, 38, 42, 71, 46, 78, 56, 49, 49 and 48 independent tumors for each column from left to right, respectively.

Source data are provided as a Source Data file.

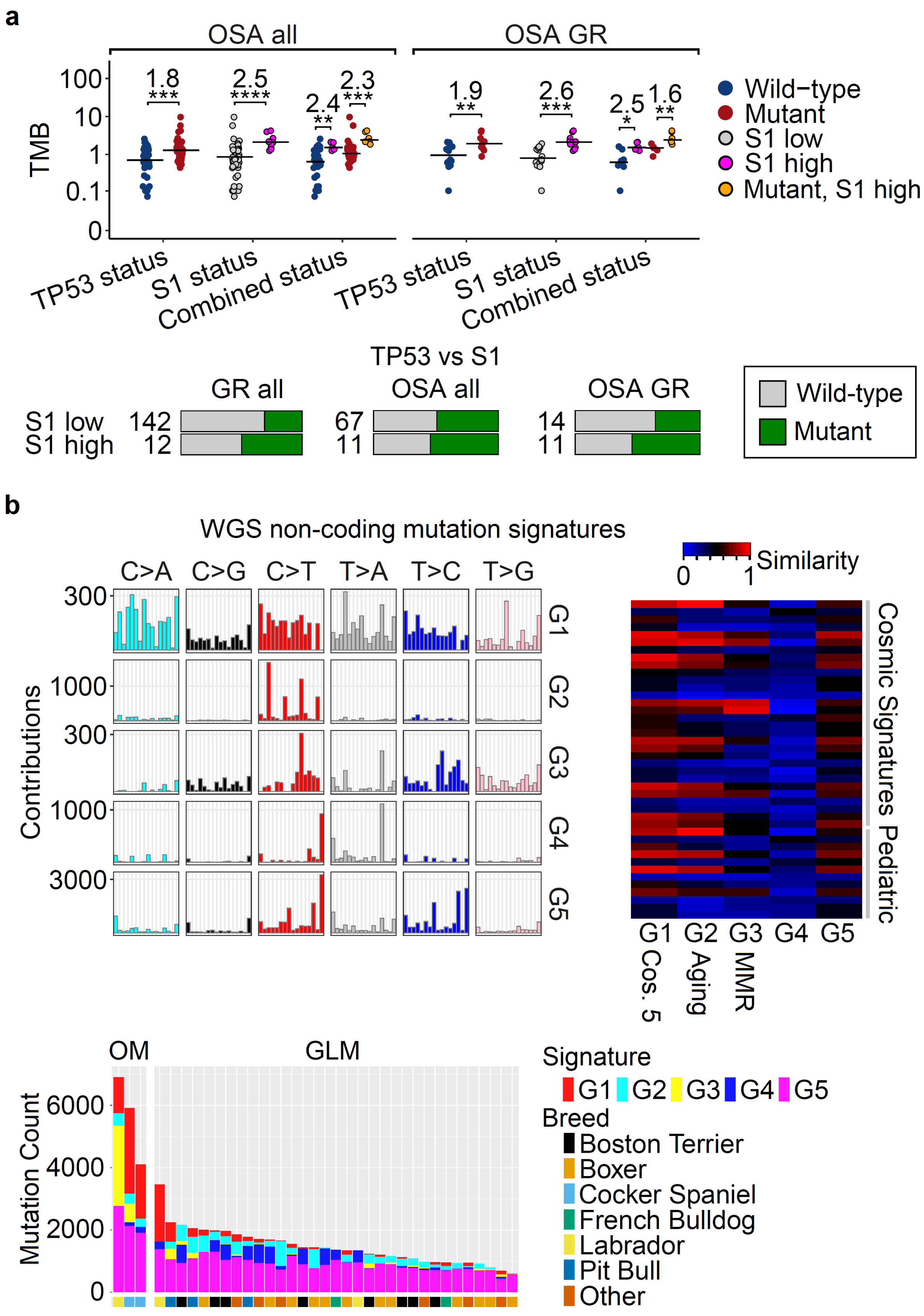
a
OSA all
OSA GR
TMB
100
10
1
0.1
0
1.8
***
2.5
****
2.4
**
2.3
***
1.9
**
2.6
***
2.5
*
1.6
**
TP53 status
S1 status
Combined status
Wild−type
Mutant
S1 low
S1 high
Mutant, S1 high
TP53 vs S1
GR all
OSA all
OSA GR
S1 low
S1 high
142
12
67
11
14
11
Wild-type
Mutant
b
WGS non-coding mutation signatures
C>A
C>G
C>T
T>A
T>C
T>G
Contributions
300
1000
3000
G1
G2
G3
G4
G5
Similarity
0
1
Cosmic Signatures
Pediatric
Cos. 5
Aging
MMR
OM
GLM
Mutation Count
6000
4000
2000
Signature
Breed
Boston Terrier
Boxer
Cocker Spaniel
French Bulldog
Labrador
Pit Bull
Other

Supplementary Figure 8. S1 mutation signature is *TP53* mutation-independent, and noncoding mutation signature in WGS tumors.

a. S1 mutation signature has a stronger association with TMB, compared to *TP53* mutation, within osteosarcoma in general (OSA all) and osteosarcoma Golden Retriever cases (OSA GR) (top). S1 is independent of *TP53* mutation (bottom). n = 39, 39, 67, 11, 34, 5, 33, 6, 14, 11, 14, 11, 9, 5, 5 and 6 independent tumors from left to right. Two-sided Wilcoxon tests were conducted, with ***: $p = 0.0009$; ****: $p = 2.2e\text{-}5$; **: $p = 0.003$; ***: $p = 0.0004$; **: $p = 0.003$; ***: $p = 0.0003$; *: $p = 0.02$ and **: $p = 0.009$ from left to right.

b. Noncoding mutation signatures detected in 36 tumors with WGS data passing our QC measures. The top two plots are presented as described in Figure 8a, while the bottom plot indicates the mutation signature distribution in each tumor, with respective breeds indicated.

Source data are provided as a Source Data file.

1 Wong, K. *et al.* Cross-species genomic landscape comparison of human mucosal melanoma with canine oral and equine melanoma. *Nat Commun* **10**, 353, doi:10.1038/s41467-018-08081-1 (2019).

2 Kim, T. M. *et al.* Cross-species oncogenic signatures of breast cancer in canine mammary tumors. *Nat Commun* **11**, 3616, doi:10.1038/s41467-020-17458-0 (2020).
