# Supplementary material for: Canine tumor mutational burden is correlated with TP53 mutation across tumor types and breeds
Source: Nat Commun. 2021 Aug 3;12:4670. doi: 10.1038/s41467-021-24836-9 (PMC8333103; doi:10.1038/s41467-021-24836-9)
Supplement: Supplementary file 3 — Description of Additional Supplementary Files [file 41467_2021_24836_MOESM3_ESM.pdf]

Description of Additional Supplementary Files

File Name: Supplementary Data 1
Description: Source, breeds, sequence quality control of published canine tumour WES and WGS data.

File Name: Supplementary Data 2
Description: Breed-specific germline base substitutions and small indels

File Name: Supplementary Data 3
Description: Canine somatic mutations, copy number alterations (CNAs), and their significance levels.

File Name: Supplementary Data 4
Description: Canonical cancer pathway genes, and human data source.

File Name: Supplementary Data 5
Description: Problematic gene list, association between TMB and gene mutations or pathway alterations, and human TMB data source.

File Name: Supplementary Data 6
Description: Canine coding and noncoding mutation signature discovery with WES and WGS data.

File Name: Supplementary Software 1
Description: Canine breed validation and prediction software v1.0.
