# Supplementary material for: Canine tumor mutational burden is correlated with TP53 mutation across tumor types and breeds
Source: Nat Commun. 2021 Aug 3;12:4670. doi: 10.1038/s41467-021-24836-9 (PMC8333103; doi:10.1038/s41467-021-24836-9)
Supplement: Supplementary file 10 — Supplementary Software 1 [file 41467_2021_24836_MOESM10_ESM.zip › Supplementary Software 1/User Manual.pdf]

Canine breed validation and prediction tools using Whole Exome Sequencing data

Author: Burair Alsaihati

Institute: University of Georgia

Version: 1.0

Date: 12/30/2020

Correspondence: burair.alsaihat25@uga.edu, burair_99@yahoo.com, szhao@uga.edu

System and installation requirements

This package of software uses a combination shell, java and R codes. Each step indicates exactly what tools or packages are needed for running. Here we summarize general requirements:

1. GNU/Linux commands and tools including sort, cat, gzip and awk
2. Java Runtime Environment 1.8 or later
3. R (the code was tested on 3.5.1 but it should work on most 3.x.x versions)
4. GATK 3 (the code was tested on 3.8.1 but should work on most 3.x.x versions)
5. ANNOVAR (ANNOtate VARiation Perl package version 2017Jul16). Please find the required Perl version for ANNOVAR in the original software documentation.

System requirements may vary depending on the amount of data you run. In our data, a RAM of 64 GB is required where many of the shell scripts (including the Java commands) are run. For the R code, a regular 8 GB RAM was sufficient to run the software on ~700 pairs of normal and tumor samples. If your dataset is much larger, larger RAM might be needed.

For installation time, please refer to each of the packages mentioned above.

Pipeline for combining germline variants and their related information

This pipeline allows the user to start from the realigned bam files for the WES normal and tumor samples in order to combine all germline variants observed in both a normal and a tumor samples of at least one case. Then extracts the variant allele frequency (VAF) data for every sample at each of the germline variants, in addition to the read coverage for every sample at each of the germline variants.

Step 1: Making an interval list file for the coding sequence (CDS) regions in the reference genome. This file is required for GATK DepthOfCoverage tool in the next step to calculate the read coverage for every base inside the CDS regions.

- Run the linux command inside the shell script make_CDS_interval_list.sh.
- Estimated runtime: 0-5 seconds
- Required software: GNU/linux awk
- Required input files: A GTF format genome annotation file
- Output files: interval_list file (see `sample_files/Canis_familiaris.CanFam3.1.99.gtf-chr1-38X-CDS.interval_list`). This is only a sample file. It does not contain all intervals for CDS regions.

Step 2: Depth of coverage calculation, germline variant calling, variant filtration and variant annotation

- For each WES normal and tumor sample, run the commands inside DepthOfCov_HaplotypeCaller_published.sh.
- Estimated runtime for a real WES normal or tumor sample with ~130x coverage (can be run in parallel if hardware resources are available): 10 hours
- Required software: GATK 3 (tested on 3.8.1), ANNOVAR (ANNOtate VARiation Perl package version 2017Jul16) and an in-house-developed java package: Pancancer.jar (provided with this package)
- Required input files:
    - An indexed fasta format reference genome
    - A GTF format genome annotation file
    - an interval list file for coding sequence (CDS) regions (generated earlier by make_CDS_interval_list.sh)
    - A realigned bam file for the WES sample
- Output files
    - A BED format file for read coverage of all bases inside CDS regions output by GATK DepthOfCoverage tool. See `sample_files/SAMN03436658_DepthofCoverage_CDS.bed`. **Most files inside `sample_files` directory are partial and do not contain all records.**
    - A VCF format file for germline variants called by GATK HaplotypeCaller and filtered by VariantFiltration. See `sample_files/SAMN03436658_rg_added_sorted_dedupped_removed.realigned.bam.filter.vcf`.
    - An annotated file for filtered germline variants output by ANNOVAR with gene names added by java class util.AddGeneNameCustomized inside Pancancer.jar. See `sample_files/SAMN03436658_rg_added_sorted_dedupped_removed.realigned.bam.filter.vcf-PASS-avinput.exonic_variant_function_WithGeneName`.

Step 3: Preparing input files to extract information for all germline variants

- Once step 2 is completed for all WES samples, each tumor and normal sample must have three essential files as described in the output files of step 2.
- You will need to prepare two files for step 4:
    - ANNOVAR file list: a tab-delimited file containing one row per normal or tumor sample. Each row has three columns: sample id (tumor or normal), sample name (case id; must be the same for normal and tumor samples of the same case) and path to ANNOVAR output with gene names. See `sample_files/annovar_file_list.txt`
    - VCF file list: a tab-delimited file containing one row per normal or tumor sample. Each row has three columns: sample id (tumor or normal), path to VCF file containing filtered germline variants and path to BED-format base coverage. See `sample_files/vcf_file_list.txt`

Step 4: Combining germline variants and extracting their VAF and read coverage information

- Finally, run the commands inside combine_germline_variants.sh to combine the germline variants in one file, extract their VAF and coverage information from each sample.
- Estimated runtime for all ~700 pair of normal and tumor WES samples (cannot be run in parallel): 27 hours
- Required software: An in-house-developed java package: Pancancer.jar (provided with this package) and GNU/linux commands (cat, sort and gzip).
- Required input files:
    - ANNOVAR file list: prepared as described in step 3
    - VCF file list: prepared as described in step 3
- Output files:
    - Sorted variant list file: A sorted combined list of all germline variants observed in both a normal and a tumor sample of at least one case. This file contains 7 columns per variant (gene, chromosome, position, reference allele, alternative allele, protein change and consequence). See `sample_files/variant_list.txt.sorted`
    - VAF matrix: A tab-delimited file with a combined list of sorted germline variants (one row per variant) and the VAF in each normal and tumor sample (one column per sample). See `sample_files/germline_VAF_matrix.txt.gz`
    - Depth matrix: A tab-delimited file with a combined list of sorted germline variants (one row per variant) and the depth values (total depth from DepthOfCoverage, total depth DP and allele depth AD from HaplotypeCaller) in each normal and tumor sample (one column per sample). See `sample_files/germline_depth_matrix.txt.gz`

For more information about using the in-house developed java tools inside Pancancer.jar that are used in this pipeline, see `AddGeneNameCustomized.ReadMe`, `CreateVariantList.ReadMe`, `GetVariantInfoFromVCF.Readme` and `AddDepthOfCoverage.Readme`

Pipeline for breed-specific variant discovery and breed validation/prediction

This pipeline allows the user to start from VAF matrix and depth matrix for each germline variant in each sample to perform breed-specific variant discovery, breed validation and/or prediction.

Step 1: Preparing input files

- Prepare a tab-delimited meta data file for the WES normal and tumor samples. See `sample_files/breed_prediction_metadata.txt`. You do not need all the columns in this file. The required columns are:

    - Sample_id: The id for the normal or tumor WES sample
    - SampleName: The case id (both normal and tumor samples of the same case must have the same case id)
    - DiseaseAcronym: An abbreviation of the tumor or cancer type
    - Breed: The breed label as assigned in the study, Mixed if not a pure breed or NA if missing
    - Status: Either Normal (if a normal sample) or Tumor (if a tumor sample).
- Run the R-code inside germline_VAF_reset_low_coverage.R to prepare a new VAF matrix file where VAF values are set to NA if the base coverage is low at the germline locus. This step is necessary to ensure minimize the discovery of artifactual breed-specific variants resulting from low coverage samples at some variant loci. **You will need to modify paths to input files and output files**.
    - Estimated runtime for all ~700 pair of normal and tumor WES samples: 2 minutes
    - Required input files:
        - VAF matrix: generated in step 4 of the first pipeline
        - Depth matrix: also generated in step 4 of the first pipeline
    - Output files:
        - VAF matrix with NA values at low coverage loci. See `sample_files/germline_VAF_matrix.reset_low_coverage.txt.gz`

Step 2: Breed-specific variants discovery

- Run the R-code inside breed_specific_variants.R to find breed-specific variants. **You will need to modify paths to input files and output files as well as the list of examined breeds**.
- Estimated runtime for ~600 normal WES samples that passed sequencing quality control: 1-2 hours
- Required code files: build_sample_meta_data.R (contains utility functions called inside the breed-specific variant discovery).
- Required input files:
    - Sample meta data file: prepared as described in step 1
    - VAF matrix with NA values at low coverage loci: generated as described in step 1
- Output files:
    - Breed-unique variants: A list for variants that are unique to a single breed. See `sample_files/breed_unique_variants.txt`
    - Breed-enriched variants: A list for variants that are enriched in one breed compared to every other breed. See `sample_files/breed_enriched_variants.txt`. This file might include all variants identified as breed-unique, in addition to more variants.
    - Breed-specific variants: A combined list for breed-specific variants identified as breed-unique or breed-enriched. See `sample_files/all_breed_specific_variants.txt`

Step 3: Breed validation and prediction

- Run the R-code inside breeds_joint_heatmap.R to perform hierarchical clustering and display the results in a heatmap image. **You will need to modify paths to input files and output files as well as the list of examined breeds and cancer types (given by DiseaseAcronym in the meta data file)**.
- Estimated runtime for ~600 normal WES samples that passed sequencing quality control: 20 minutes

- Required code files: build_sample_meta_data.R (contains utility functions called inside the breeds_joint_heatmap.R).
- Required input files:
  - Sample meta data file: prepared as described in step 1
  - VAF matrix with NA values at low coverage loci: generated as described in step 1
  - Breed-specific variants: A combined list for breed-specific variants generated in step 2
- Output files:
  - Breed validation heatmap: A heatmap for VAF values of breed-specific variants in normal samples displayed after hierarchical clustering is performed and breed labels displayed at the top. Samples failing breed validation will cluster outside the largest cluster of their own breed. See `sample_files/breeds_heatmap_main_305_dpi.png`
  - Breed prediction heatmap: A heatmap for VAF values of breed-specific variants in normal samples displayed after hierarchical clustering is performed and breed labels displayed at the top. Samples labeled as Unknown breed falling inside the main cluster of a known-breed are predicted to have the same breed. See `sample_files/breeds_heatmap_assignment_305_dpi.png`
  - Sample order file: text-format files listing all samples, in addition to their breed labels, in the same order they appeared in the heatmap. These files aim to simplify the identification of samples failing the breed validation or the samples assigned to a known breed. The actual assignment is done manually after comparing the heatmap image and the output text. See `sample_files/main_clusters.txt` and `sample_files/assignment_clusters.txt`
