# Supplementary figures and images for: Canine tumor mutational burden is correlated with TP53 mutation across tumor types and breeds
Source: Nat Commun. 2021 Aug 3;12:4670. doi: 10.1038/s41467-021-24836-9 (PMC8333103; doi:10.1038/s41467-021-24836-9)

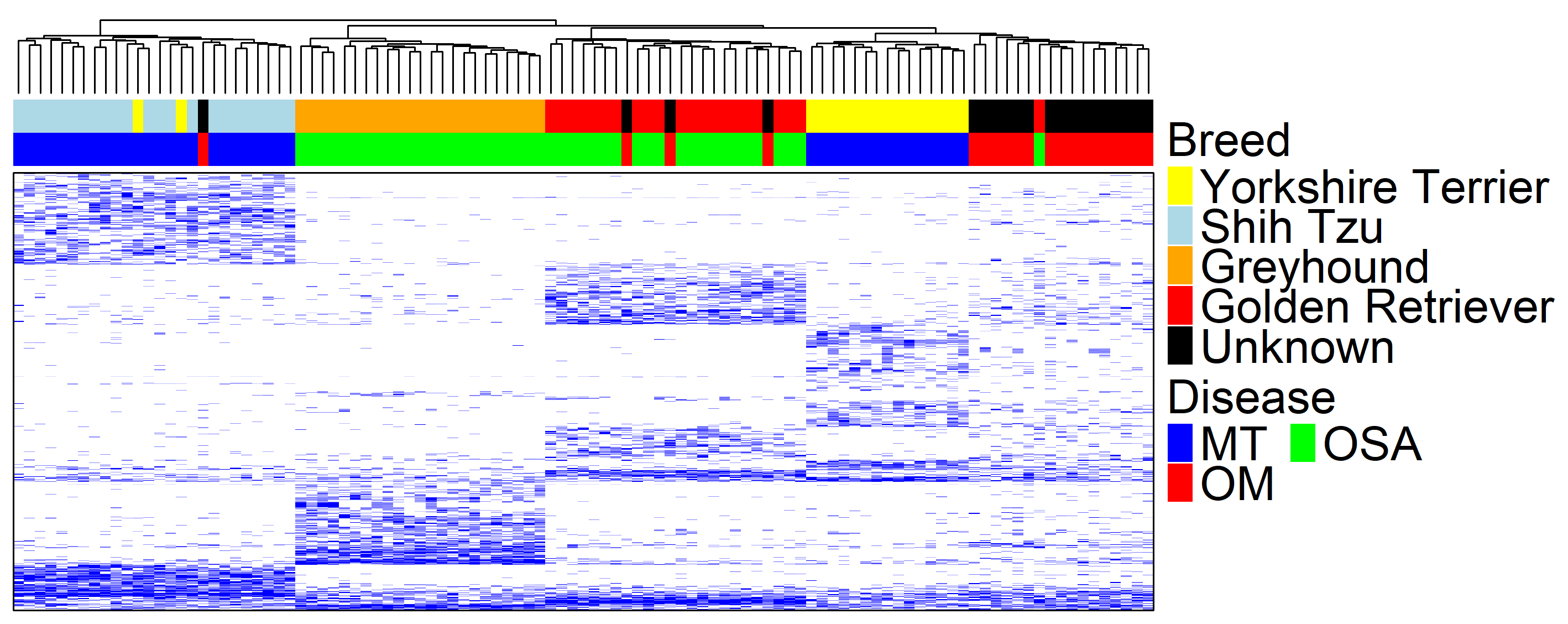

Supplement: Supplementary file 10 — Supplementary Software 1 [file 41467_2021_24836_MOESM10_ESM.zip › Supplementary Software 1/sample_files/breeds_heatmap_assignment_305_dpi.png]

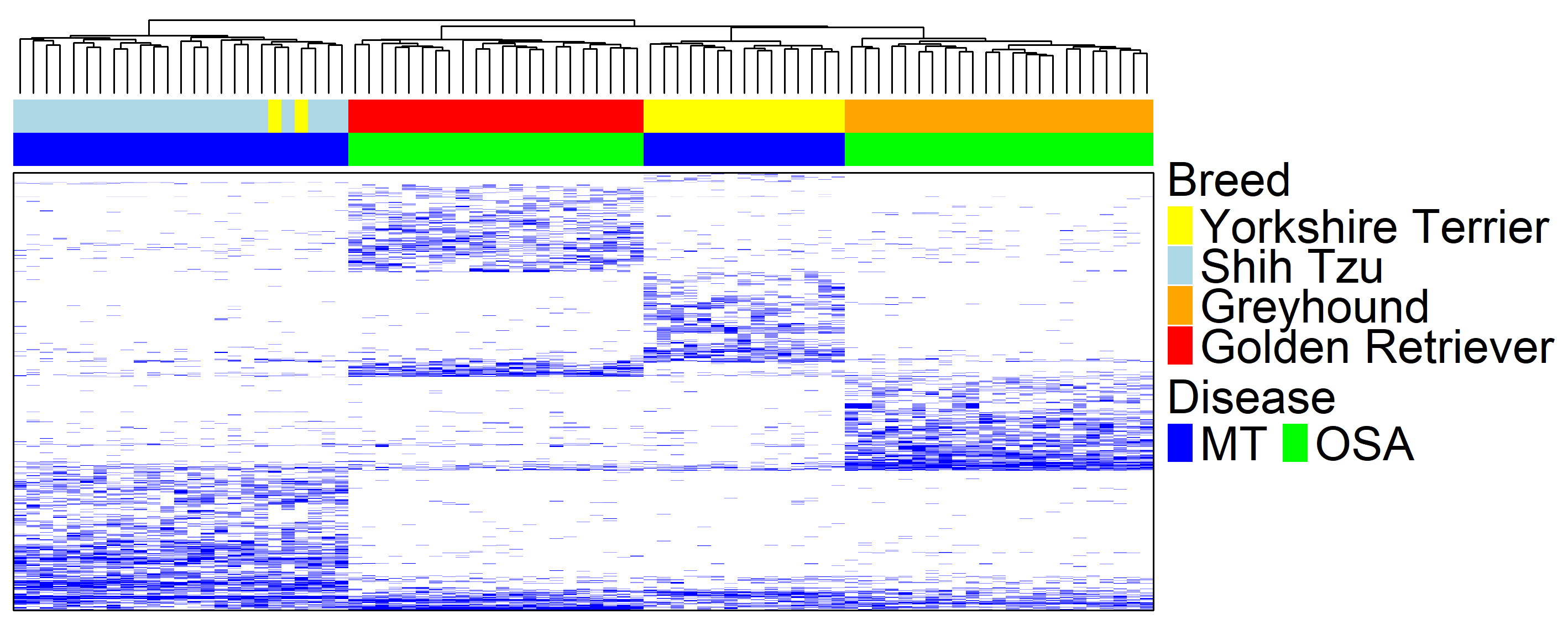

Supplement: Supplementary file 10 — Supplementary Software 1 [file 41467_2021_24836_MOESM10_ESM.zip › Supplementary Software 1/sample_files/breeds_heatmap_main_305_dpi.png]
