# Supplementary material for: Canine tumor mutational burden is correlated with TP53 mutation across tumor types and breeds
Source: Nat Commun. 2021 Aug 3;12:4670. doi: 10.1038/s41467-021-24836-9 (PMC8333103; doi:10.1038/s41467-021-24836-9)
Supplement: Supplementary file 11 — Reporting Summary [file 41467_2021_24836_MOESM11_ESM.pdf]

nature research

Corresponding author(s): Shaying Zhao

Last updated by author(s): Jun 22, 2021

# Reporting Summary

Nature Research wishes to improve the reproducibility of the work that we publish. This form provides structure for consistency and transparency in reporting. For further information on Nature Research policies, see our Editorial Policies and the Editorial Policy Checklist.

## Statistics

For all statistical analyses, confirm that the following items are present in the figure legend, table legend, main text, or Methods section.

| n/a | Confirmed | |
|---|---|---|
| ☐ | ☒ | The exact sample size (*n*) for each experimental group/condition, given as a discrete number and unit of measurement |
| ☐ | ☒ | A statement on whether measurements were taken from distinct samples or whether the same sample was measured repeatedly |
| ☐ | ☒ | The statistical test(s) used AND whether they are one- or two-sided<br>*Only common tests should be described solely by name; describe more complex techniques in the Methods section.* |
| ☒ | ☐ | A description of all covariates tested |
| ☐ | ☒ | A description of any assumptions or corrections, such as tests of normality and adjustment for multiple comparisons |
| ☐ | ☒ | A full description of the statistical parameters including central tendency (e.g. means) or other basic estimates (e.g. regression coefficient) AND variation (e.g. standard deviation) or associated estimates of uncertainty (e.g. confidence intervals) |
| ☐ | ☒ | For null hypothesis testing, the test statistic (e.g. *F*, *t*, *r*) with confidence intervals, effect sizes, degrees of freedom and *P* value noted<br>*Give P values as exact values whenever suitable.* |
| ☒ | ☐ | For Bayesian analysis, information on the choice of priors and Markov chain Monte Carlo settings |
| ☒ | ☐ | For hierarchical and complex designs, identification of the appropriate level for tests and full reporting of outcomes |
| ☐ | ☒ | Estimates of effect sizes (e.g. Cohen's *d*, Pearson's *r*), indicating how they were calculated |

*Our web collection on statistics for biologists contains articles on many of the points above.*

## Software and code

Policy information about availability of computer code

| | |
|---|---|
| Data collection | SRA-Toolkit v2.9.1 |

Data analysis

In-house software package:
Breed prediction v1.0, deposited to GitHub at https://github.com/ZhaoS-Lab/breed_prediction

Publicly available software:
ANNOVAR v2017Jul16
BWA v0.7.17
DNAcopy v1.6.4
GATK v3.8.1
GATK4 MuTect2 v4.1.6
Picard v2.16.0
Lofreq v2.1.2
MuTect v1.1.7
R package statmod v1.4.36.
SAMTools v1.9
SEG v1.0.0
Somaticseq v3.4.1
Strelka v2.9.2
SignatureAnalyzer version year 2017, Broad Institute, open source
VarScan v2.4.2

For manuscripts utilizing custom algorithms or software that are central to the research but not yet described in published literature, software must be made available to editors and reviewers. We strongly encourage code deposition in a community repository (e.g. GitHub). See the Nature Research guidelines for submitting code & software for further information.

## Data

Policy information about availability of data

All manuscripts must include a data availability statement. This statement should provide the following information, where applicable:

- Accession codes, unique identifiers, or web links for publicly available datasets
- A list of figures that have associated raw data
- A description of any restrictions on data availability

Our study focuses on publicly available canine and human data. Canine WES and WGS data were downloaded from the Sequence Read Archive (SRA) database, as listed in Table S1A. We also obtained case information from relevant publications. Harmonized variant-level data are provided in Supplementary Tables. Due to their large size, harmonized read-level data cannot be submitted as Supplementary Data; however they will be available upon request.

Mutated or amplified/deleted genes, and altered pathways in human cancers were extracted from published studies, as described in the Article and Supplementary Tables.

Source data are provided with this paper, in relevant Supplementary Tables.

# Field-specific reporting

Please select the one below that is the best fit for your research. If you are not sure, read the appropriate sections before making your selection.

[x] Life sciences [ ] Behavioural & social sciences [ ] Ecological, evolutionary & environmental sciences

For a reference copy of the document with all sections, see nature.com/documents/nr-reporting-summary-flat.pdf

# Life sciences study design

All studies must disclose on these points even when the disclosure is negative.

Sample size

Sample size was determined by: 1) publicly available whole exome sequencing (WES) and/or whole genome sequencing (WGS) data of canine tumors; and 2) our sequence data quality control analyses. We have performed power calculations to indicate that the sample size is large enough to support our major findings, as illustrated in Figure S5 and described in the article.

Data exclusions

We excluded canine cases whose WES and WGS data failed our comprehensive sequence quality control measures, as shown in Figures 1 and S1. Furthermore, for breed-related analyses, we excluded animals whose breeds cannot be validated, corrected or predicted, as illustrated in Figure 2.

Replication

For our breed validation/prediction strategy which uses the WES dataset, we used the WGS dataset for validation (Figures 2 and S2). For mutation discovery, we compared our findings with those from the original publications (Figure S4). For TMB conclusions, we used different software tools (Figure S7a). All discovery and validation analyses are performed independently. All major findings are successfully confirmed.

Randomization

This is not relevant to our study, because our study has been performed by combing all samples from 11 independent studies after rigorous quality control and data harmonization. No sample allocations into experimental groups is involved.

| Blinding | Blinding was not relevant to our study, as no sample allocations into experimental groups was performed. |
|---|---|

# Reporting for specific materials, systems and methods

We require information from authors about some types of materials, experimental systems and methods used in many studies. Here, indicate whether each material, system or method listed is relevant to your study. If you are not sure if a list item applies to your research, read the appropriate section before selecting a response.

## Materials & experimental systems

| n/a | Involved in the study |
|---|---|
| ☒ | ☐ Antibodies |
| ☒ | ☐ Eukaryotic cell lines |
| ☒ | ☐ Palaeontology and archaeology |
| ☒ | ☐ Animals and other organisms |
| ☒ | ☐ Human research participants |
| ☒ | ☐ Clinical data |
| ☒ | ☐ Dual use research of concern |

## Methods

| n/a | Involved in the study |
|---|---|
| ☒ | ☐ ChIP-seq |
| ☒ | ☐ Flow cytometry |
| ☒ | ☐ MRI-based neuroimaging |
